# COMPARISON OF A FRAILTY INDEX WITH CARDIOVASCULAR RISK SCORES IN PREDICTING CARDIOVASCULAR DISEASE MORTALITY

**DOI:** 10.1093/geroni/igz038.2944

**Published:** 2019-11-08

**Authors:** Dustin S Kehler, Olga Theou, Kenneth Rockwood

**Affiliations:** Dalhousie University, Halifax, Nova Scotia, Canada

## Abstract

We compared the predictive and discriminative ability of frailty with traditional cardiovascular risk scores to estimate 10-year cardiovascular disease (CVD) mortality risk. Individuals aged 20-79 years old from the National Health and Nutrition Examination Survey who were free from CVD were included (n= 32,066). A 33-item frailty index (FI) which excluded CVD and diabetes-related variables was calculated. We calculated the Framingham Disease Risk (FDR) Hard Coronary Heart Disease and General CVD risk scores, the American Heart Association/American College of Cardiology (AHA/ACC) atherosclerotic cardiovascular disease risk equation, and the European Systematic Coronary Risk Estimation tool. A total of 322 individuals died (1.0%) from CVD. There was a low correlation between the FI and CVD risk scores (spearman’s r= 0.19-0.33; p<0.0001) and a weak to strong correlation between CVD risk scores (spearman’s r=0.19-0.88; p<0.0001). The competing-risks hazard ratio for CVD mortality for every 1% increase in the FI was 1.040 (95% CI: 1.032-1.048; p<0.0001) in an age and sex-adjusted model. The FI was independently predictive of CVD mortality when the other CVD risk scores were added to the model. The area under the receiving operating characteristic (ROC) curve was 0.800 (95% CI: 0.789-0.808; p<0.0001) for the FI. ROC values for the CVD risk scores ranged from 0.710 (95% CI: 0.700-0.721; p<0.0001) for the AHA/ACC risk score to 0.779 (95% CI: 0.770-0.789; p<0.0001) for the FDR General CVD risk score. An FI calculated with non-CVD and diabetes variables can predict 10-year CVD mortality risk independently of traditional CVD risk scores.

